# Oral cancer among Khat users: finding evidence from DNA analysis of nine cancer-related gene mutations

**DOI:** 10.1186/s12903-021-01981-7

**Published:** 2021-12-07

**Authors:** Sawsan Abdullah Alshahrani, Wedad Saeed Al-Qahtani, Nawaf Abdulrahman Almufareh, Dalia Mostafa Domiaty, Gadah Ibraheem Albasher, Fatmah Ahmed Safhi, Fatima Abdullah AlQassim, Mashael Alhumaidi Alotaibi, Tahani Mohamed Al-Hazani, Bassam Ahmed Almutlaq

**Affiliations:** 1 Ministry of National Guard and Health Affairs, Riyadh, Saudi Arabia; 2 Department of Forensic Sciences, College of Criminal Justice, Naif Arab University for Security Sciences, P.O. Box 6830, 11452 Riyadh, Saudi Arabia; 3 Department of Paediatric Dentistry and Preventive Dental Sciences, Riyadh Elm University, Riyadh, Saudi Arabia; 4 College of Science, Department of Biology, University of Jeddah, P.O. Box 13151, 21493 Jeddah, Saudi Arabia; 5 Department of Zoology, College of Science, King Saud University, Riyadh, Saudi Arabia; 6 Department of Biology, College of Science, Princess Nourah bint Abdulrahman University, Riyadh, Saudi Arabia; 7 Regional Laboratory and Blood Bank, Dammam, Saudi Arabia; 8 Department of Biology, College of Science, Jouf University, Sakakah, Saudi Arabia; 9 Department of Biology, College of Sciences and Humanities, Prince Sattam Bin Abdulaziz University, P.O. Box 83, 11940 Al-Kharj, Saudi Arabia; 10 College of Medicine, University of Hail, Hail, Saudi Arabia

**Keywords:** Oral squamous cell carcinomas, Khat users, DNA analysis, Cancer-related gene, Mutations

## Abstract

**Background:**

Khat leaves contain the alkaloid cathinone. Research shows that khat might provoke toxicity, mutagenicity, as well as carcinogenicity.

**Methods:**

Two groups were identified as khat abusers and were categorized by abuse time and diagnosis of oral squamous cell carcinoma (OSCC). Here, 41 participants from Group 2 were short-term khat users, and 42 participants were long-term khat users. The control group included 30 healthy individuals.

The coding exons included nine cancer-related genes and were analysed. The histopathological research was conducted with H&E staining along with the TP53 protein expression by implementing immunohistochemical analyses.

**Results:**

Here, 41 short-term khat users carried seven somatic mutations in four out of nine cancer-related genes: 29/41(70.73%) *ARID1A*, 24/41(58.53%) *MLH1*, 34/41(82.92%) *PIK3CA* and 36/41(87.80%) *TP53*. The 42 long-term khat users incorporated nine somatic mutations in five out of nin ecancer-related genes: 40/42(95.23%) *ARID1A*, 36/42(85.71%) *ARID2*, 29/42(69.04%) *PIK3CA*, 27/42(64.28%) *MLH1*, and 35/42(83.33%) *TP53*. Every khat user had somatic mutations related to OSCC affecting the gingiva and the lower lip. TP53 protein expression was confirmed in all immunohistochemical oral tests. Carcinoma was also positive in the histopathological analysis.

**Conclusions:**

Khat is a mutagenic and carcinogenic plant that provoked OSCC among short-term khat users (<15 years of use) and long-term users (>15 years of use).

## Background

Oral squamous cell carcinoma (OSCC) is one of the most prevalent variations of cancer with high rates of spread in Asia [1–4]. Some epidemiological researches shows that with gradual reduction of oral cancer’s incidences in general, lethal outcomes related to OSCC have also markedly declined. In fact, this can be explained by improved lifestyle management, new opportunities for timely screening, and general progress of healthcare [5]. The frequency of OSCC is increasing because of risk factors associated with Middle Eastern populations [6]. Indeed, tobacco smoking is increasing in the Middle East [7]. Squamous cell carcinomas represent a wide spectrum of pathologies that can appear in tissues that serve as formal body barriers (oral cavity, oesophagus, lungs, airways, vulva, cervix, urethra, and human skin); they protect people from external environmental effects [8].

*Catha Edulis Forsk* or simply khat is a natural plant that belongs to the *Ceastraceae* family. Its leaves and sprouts can be chewed due to its similarity to amphetamines in the context of biology and associated stimulating reactions [9, 10]. Khat use is a cultural tradition and is used in different countries from the Middle East to Eastern Africa especially Saudi Arabia, Yemen, Kenya, and Eritrea. The khat’s leaves are commonly chewed at social venues. Fresh leaves are more prioritized for chewing including swallowing the liquid of the plant. The micro leftovers from the chewed plant typically stay in the mouth’s buccal sulcus in a unidirectional or bipartite position. If the leftovers stay in these positions for several hours, they still produce integral chemical elements [9, 11, 12]. Stimulants such as cathinone and cathine are included in khat’s biology, which explains why chewing the plant influences the user’s cognitive functions and emotional state.

Khat is a typical neurotransmitter that launches parasympathetic activity, thus resulting in the inevitable release of acetylcholine [12]. Research has shown that khat stimulates active release of a stress hormone known as norepinephrine and increasing the user’s alertness and overall vigilance [9, 12, 14]. Like any other neurotransmitter operating through acetylcholine receptors, khat leads to neurological disorders and increases the risk of cancer [12, 13]. Recent studies have demonstrated that nerves from the peripheral group (sympathetic, parasympathetic, and sensory) affect the emergence of tumours and activity of stromal cells, which may trigger the development of diverse physical and haematological pathologies and malignancies [13–17].

The current research examines short-term and long-term outcomes of using khat in male participants diagnosed with and without OSCC. Examination is completed by identifying somatic mutations via analysis of targeted gene panels including sequencing. This study also assessed the histopathological changes and possible immunohistochemical expressions among short-term (2 – 5 years) and long-term (above 5 years) users.

## Methods

### Human participants and ethical compliance

 The study was conducted in accordance with the standards of the Deanship of Scientific Research for Princess Nourah Bint Abdulrahman University. The local ethics committee from KACST, Riyadh, Saudi Arabia approved the study (number H-01-R059, IRB LOG number 20-0242). In addition, all patients voluntarily provided written informed consent and released their samples for research purposes.

###  Respondents and clinical analysis

This study was retrospective research and covered 122 male participants to evaluate short-term (2–5 years) and long-term (above 5 years) outcomes of using khat in male participants diagnosed with and without OSCC between October 2020 - May 2021 in the Surgery & Dental/Maxillofacial Clinics, Histopathology and Biochemistry Laboratories at King Fahad Medical City, Riyadh, KSA. Medical history and reports of all patients were assessed to obtain demographic input and medical parameters.

#### Exclusion criteria

Exclusion criteria were a long history of smoking (above 10 years), chronic illnesses (for instance, diabetes and/or hypertension), and records of any cancer in family history (except khat usage as a reason).

#### Inclusion criteria

All patients who used khat before chemotherapy/radiotherapy and the ones diagnosed with OSCC were included in the final list. Their age varied between 51 and 70, and they fit the study’s criteria. In addition, healthy participants were selected for the control group with age ranging from 25 to 35 years old.

#### Study design and clinical analysis

This study included 113 participants in total were selected for the study and divided into three groups. The first category had 30 persons, i.e., control group with healthy individuals without any khat use. Patients who chewed khat were further distributed by groups based on duration of use and clinical analysis. In the second group, participants with short-term use of khat and OSCC diagnosis were included 41 patients. The third group included participants with long-term use of khat and OSCC diagnosis for a total of 42 patients. Table 1 below describe the study’s groups in detail.

**Table 1:** Demographic data on the participants

Covariate	Group 1: control patients	Group 2: short-term khat users diagnosed with OSCC	Group 3: long-term khat users diagnosed with OSCC
No. of participating patients	30	41	42
Age during diagnosis			
< 40 years	13	22	24
≥ 40 years	17	19	18
The amount of khat used			
Khat abuse patterns (times of use per week)	–	2–3	4–6
The consumption time (in years)	–	15–20	> 20
Cancer in family history (not provoked by khat)	–	–	–
History of Smoking	–	28/ 41 (41.46%)	19/ 42 (45.23%)
Hypertension (HTN)	–	28/41 (68.29%)	37/42 (88.09%)
Diabetes mellitus (DM)	–	24/41 (58.53%)	29/42 (69.04%)

### Collection of samples

#### DNA extraction and blood samples

DNA extraction was performed using fresh peripheral blood samples (before surgery) from all the patients and healthy participants utilizing Qiagen DNA isolation kit (Catalogue Number 69,506, Quigen, Hilden, developed in Germany) according to the manufacturer’s instructions. The DNA samples’ concentration and quality were measured using a NanoDrop Spectrophotometer from Thermo Scientific (United States).

##### Targeted panel sequencing’s capture

Nine cancer-related genes was analysed: *ARID1A*, *ARID2*, *TP53*, *NUMA1*, *CREBBP*, *NCOA2*, *PPP2R1A*, *MLH1*, and *PIK3CA*. Sixty nanograms of DNA were reinforced by applying three associations of 502 primer pairs with a Ion AmpliSeq Comprehensive Cancer Panel developed by *Life Technologies* to cover each coding exon over 9 cancer-associated genes. Amplicon ligation used barcoded adaptors provided by Ion Amplicon Library Kit (the manufacturer is Life Technologies). Barcoded catalogues were further combined with sequencing beads by ensuring the prepared PCR emulsion. They were also amplified with a IonChef in compliance with Ion Torrent instructions (developed and provided by Life Technologies). The fragment analyser (AATI) and Qubit (Invitrogen) were selected as choices to define quality and the numerical value of enriched catalogues. The sequencing procedure was conducted using the ion proton sequencer and by applying the Ion PI chip in accordance with the manufacturer’s instructions.

####  Immunohistochemistry and Histological Analysis

Paraffin-embedded blocks of biopsy fragments were taken from short- and long-term users of khat with OSCC. The specimens were derived during surgical operations and prior to chemotherapy/radiotherapy procedures; 3- to 5-µm sections were made for histopathology and immunohistochemical tests. Furthermore, every block could generate a pair of slides. Eosin and haematoxylin were used. The sections with stains were scanned at increased magnification (20X) to evaluate the histopathological sites of the sections selected. They were then photographed using a photomicroscope. Finally, immunohistochemical tests were completed on the other slide from every block utilizing the TP53 antibody following the protocol [18].

#### Analysis of statistics

Raw data were generated from the sequencing procedure and was applied to the Hg19 genome of reference implementing the Ion Torrent Suite, Version 4.2. The extent of scope and coverage was defined with a help of the special plugin (Torrent Coverage Analysis). Another plugin (Torrent Variant Caller Version 4.2) was used to identify short insertions/deletions (INDELs) as well as single nucleotide variants (SNVs). Additionally, the special Variant Effect Predictor (VEP) was utilized to comment every item incorporating the library from COSMIC: v.70; dbSNP 138 and 1000 Genomes: phase 1. The items with coverage/scope less than 25 and frequency below 5% were excluded. Moreover, the research used in silico tests via a PolyPhen-2 [19] as well as SIFT [20] to determine whether the selected mutations transform into dangerous or non-harmful outcomes.

Pearson’s correlation coefficient was used by SigmaStat software Version 3.5 (Systat Software) for quantitative findings and were interpreted by comparing the standard deviation and mean values. P values < 0.05 were considered to be statistically significant.

## Results

### Malicious ***ARID1A***, ***ARID2***, ***CREBBP***, ***NCOA2***, ***NUMA1***, ***PPP2R1A***, ***MLH1***, ***PIK3CA***, and ***TP53*** Gene mutations

After completing the clinical trials, nine of 92 respondents were excluded because they did not meet the study criteria. Thus, this study presented 83 participants who chewed khat: 41 participants were short-term khat users, and 42 participants were long-term khat users. All participants had somatic mutations and were diagnosed with OSCC on the gingival and lower lip areas; these conditions contrasted with the control group of 30 healthy participants. Eventually, 7 somatic mutations in four of nine cancer-related genes were identified in the short-term khat users. The incidence of somatic mutations was identified in the following order: 29/41(70.73%) *ARID1A*, 24/41(58.53%) *MLH1*, 34/41(82.92%) *PIK3CA*, and 36/41(87.80%) *TP53*. In turn, *ARID2, NUMA1*, *CREBBP, NCOA2*, and *PPP2R1A* showed no gene mutations.

On the other hand, nine somatic mutations in five of nine cancer-related genes were identified in the group of long-term khat users. Mutations were identified in the following order: 40/42(95.23%) *ARID1A*, 36/42(85.71%) *ARID2*, 29/42(69.04%) *PIK3CA*, 27/42(64.28%) *MLH1*, and 35/42(83.33%) *TP53*. There were no visible mutations in *NUMA1*, *CREBBP, NCOA2*, and *PPP2R1A* relative to the control group.

**Fig. 1:**
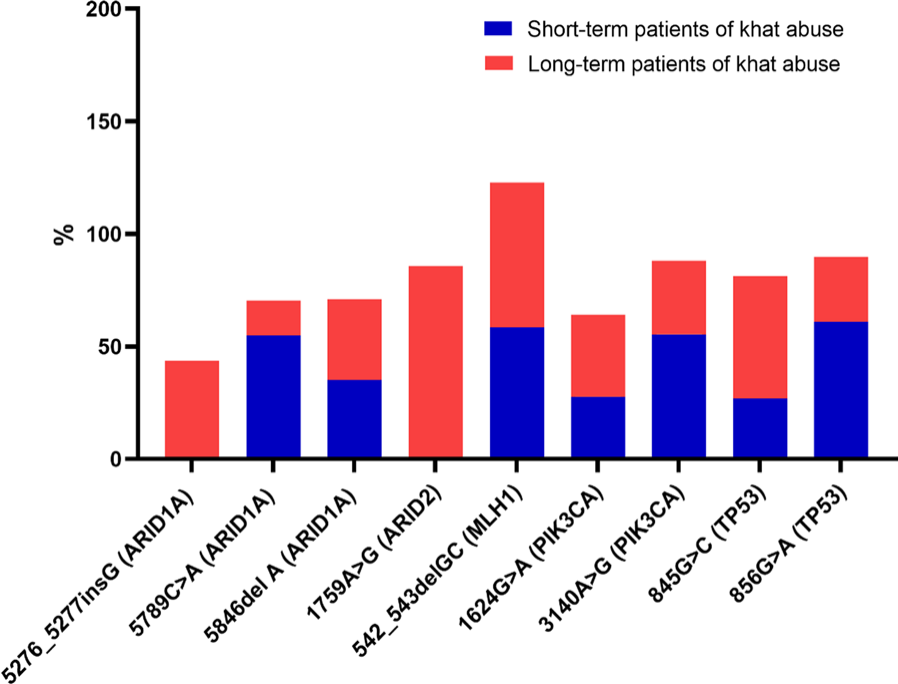
The incidence of genetic mutations for the two groups including short- and long-term khat users diagnosed with OSCC

Somatic mutations related to all observable genes are provided in Table 2 for short-term khat users. Outcomes for long-term khat users are listed in Table 3. The incidence of genetic mutations for the two groups are evaluated and presented in Fig. 1. Moreover, there are no mutations in the DNA samples from the control group.

**Table 2:** Seven somatic mutations in four of nine cancer-related genes were seen among short-term khat users diagnosed with OSCC

Gene	Chromosome	Exon	Nucleotide	Mutation type	Protein change	Previously reported in other populations
***ARID1A***	1	20/20	c.5789 C>A	Substitution	p. S1930	Yes
			c.5846del A	Deletion	p. H1949fs	Yes
***MLH1***	3	6/19	c.542_543delGC	Deletion	p. G181D	Yes
***PIK3CA***	3	9	c.1624G>A	Substitution	p. E542K	Yes
		20	c.3140 A>G	Substitution	p. H1047R	Yes
***TP53***	17	8	c.845G>C	Substitution	p. R150P	Yes
			c.856G>A	Substitution	p. E11Q	Yes

**Table 3:** Nine somatic mutations in five of nine cancer-related genes among long-term khat users diagnosed with OSCC

Gene	Chromosome	Exon	Nucleotide	Mutation type	Protein change	Previously reported in other populations
***ARID1A***	1	20/20	c.5276_5277insG	Insertion	p. E1760fs	Yes
			c.5789 C >A	Substitution	p. S1930	Yes
			c.5846del A	Deletion	p. H1949fs	Yes
***MLH1***	3	6/19	c.542_543delGC	Deletion	p. G181D	Yes
***PIK3CA***	3	9	c.1624G >A	Substitution	p. E542K	Yes
		20	c.3140 A >G	Substitution	p. H1047R	Yes
***ARID2***	12	24	c.1759 A >G	Substitution	p. S587G	Yes
***TP53***	17	8	c.845G >C	Substitution	p. R150P	Yes
			c.856G >A	Substitution	p. E11Q	Yes

## Histopathology

**Fig. 2:**
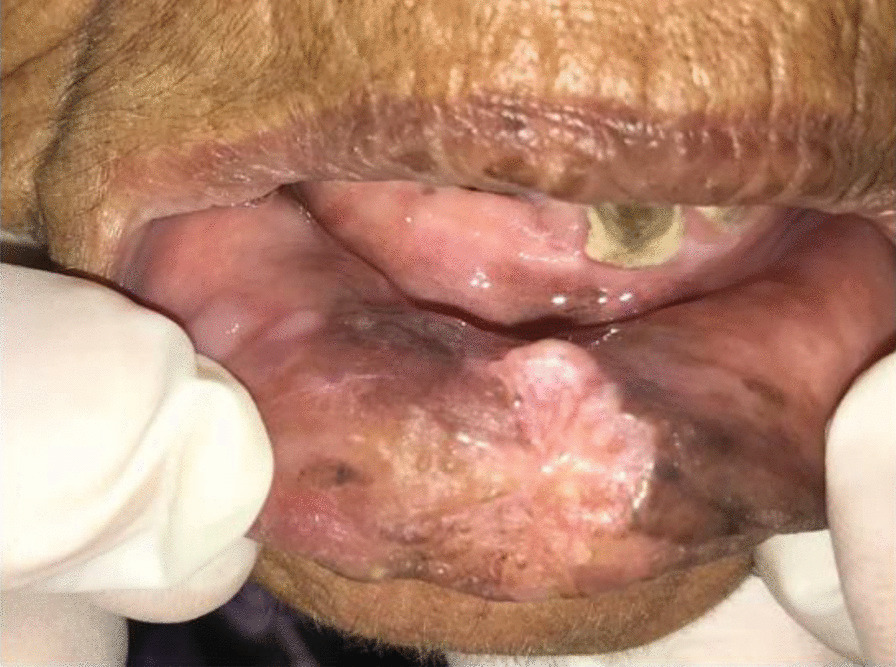
Images of a 70-year-old patient with khat chewing experience since the age of 33 (a long-term khat abuser diagnosed with OSCC on the lower lip) shows exophytic lesions (initiated two months ago). The clinical evaluation revealed the following: red-white exophytic lesion accompanied by dark discoloration, lack of sensation during palpation, history of fever, and no lymph nodes seen. Biopsy analysis revealed verrucous carcinoma on the lower lip

**Fig. 3:**
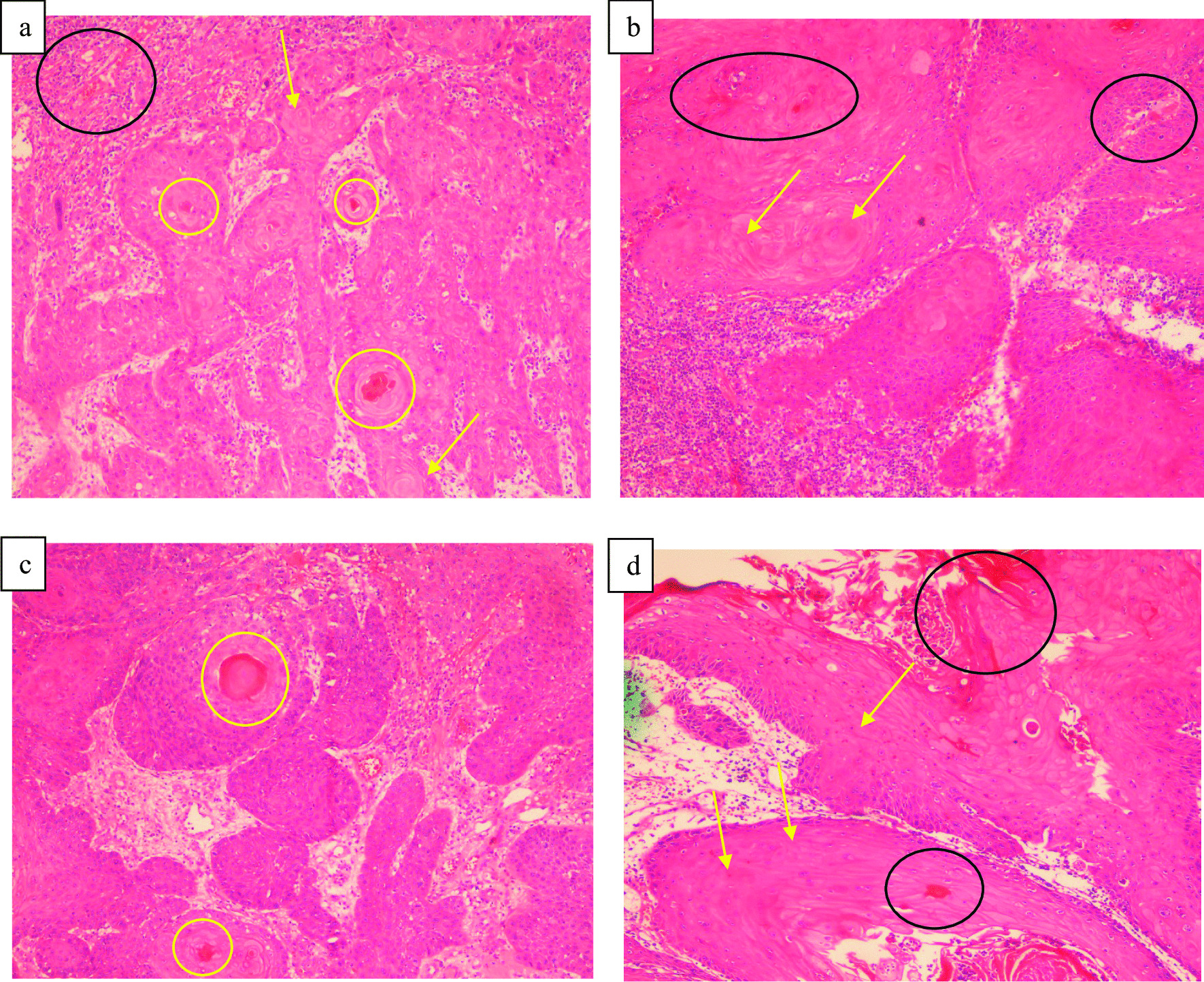
Images of micro-histopathology for H&E samples (with stain) (scale bar: 100 μm). SCC of the oral cavity areas (namely gingival and lower lip) indicates of dissymmetry among squamous cells; black circles are vascular invasion with abnormally sized cells with eminent red nucleoli (showed as yellow circles), pleomorphic big cells (related to yellow arrows), atypical mitotic effects, and some minor nucleoli. Panel **a** is gingival-related SCC, **b** is lower lip SCC related to the group of short-term khat users, **c** is gingival-related SCC, and **d** is lower lip SCC from the group of long-term khat users

Histopathological analysis involving photomicrographs based on deriving OSCC paraffin-embedded blocks of biopsy samples from affected gingival and lower lip areas. One of these paraffin-embedded blocks of biopsy samples was taken from a 70-year-old patient who had chewed khat since the age of 33 (e.g., a long-term khat abuser diagnosed with OSCC on the lower lip (Fig. 2). For the histopathological analysis, all the slides were prepared from the paraffin-embedded blocks of biopsy samples from the group of short-term khat users (Fig. 3a and b) as well as from the group of long-term khat users (Fig. 3c and d).

Poorly differentiated cells with occasional histological structures were seen. For instance, there are pathological mitotic effects, certain patterns of proliferation disintegration, necrotic activity, vessels with thin walls, vascular invasion filled with red blood cells as well as pleomorphic cells with changing forms and various sizes of nucleoli. Meanwhile, the histopathological patterns were observed in the gingival and lower lip areas (Fig. 3c and d) from the group of long-term khat users.

### Immunohistochemical findings for protein expression of TP53

**Fig. 4:**
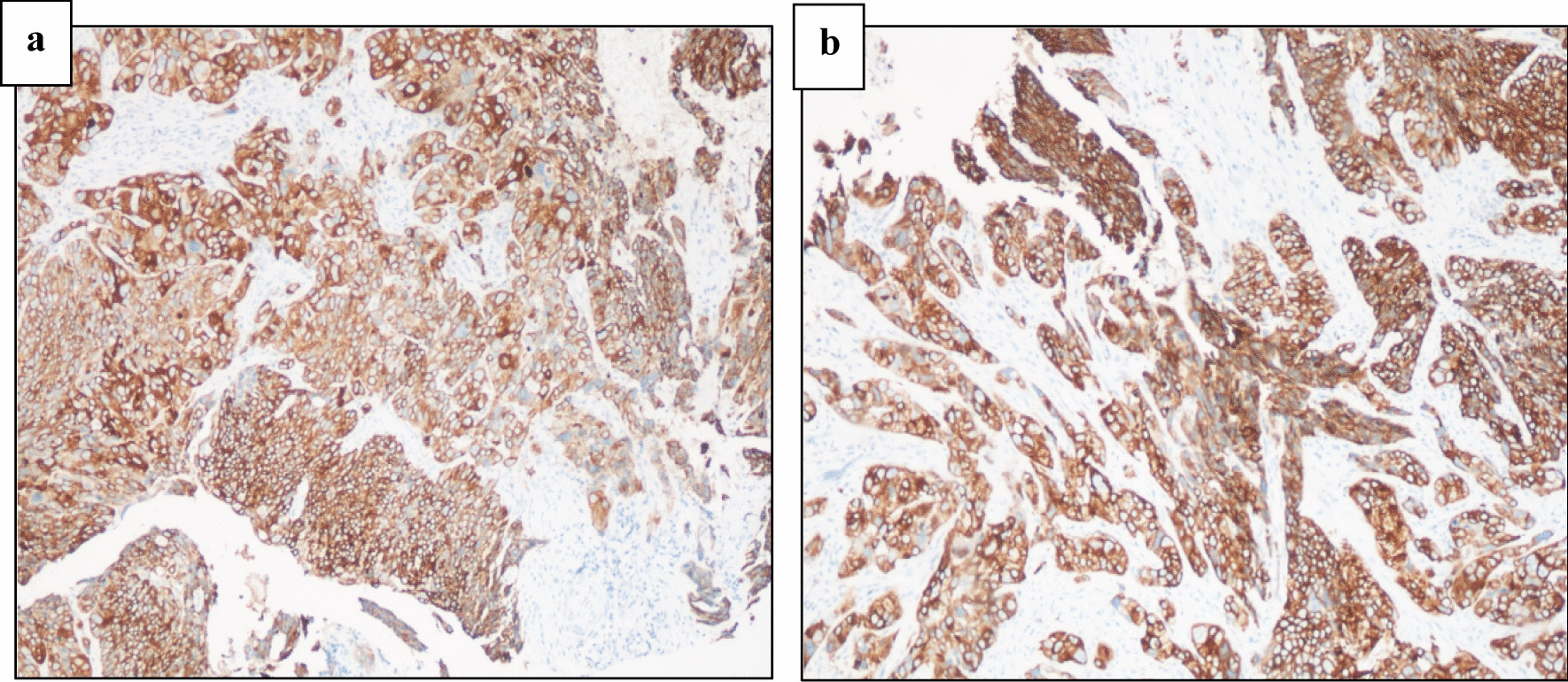
Photomicrographs of immune-stained areas applying TP53 antibody under a specific scale (100 μm). Immunohistochemical slides focused on staining with TP53 antibody. Samples showed a positive status for TP53 proteins among all khat users from the two groups. SCC of the oral cavity areas (gingival sites) was presented as follows: **a** gingival site SCC among short-term khat users; **b** gingival site SCC among long-term khat users

Expression of TP53 was investigated using immunohistochemistry analysis, which can be seen in Fig. 4. Immunohistochemistry analysis revealed mainly positive protein expression of staining with TP53 antibody in the oral area (specifically gingival sites) in the short-term khat users. Strong and moderate protein expression for TP53 were identified in the same portion of histological oral areas in the group of long-term khat users. Immune staining was identified in 20% reactions for positive interactions with the special antibodies. This is why they have been interpreted as positive reactions (Fig. 4).

 In addition, TP53 expression was identified in most oral (gingival sites) areas for both groups. Furthermore, the process of deriving antigens was computed in compliance with the most up-to-date quantitative H-scores for each situation and then by multiplying the values in relation to the following categories: more than 50% (strong), 20–40% (moderate), 1–20% (weak), and 0% (none) (Table 4).

**Table 4:** Level of OSCC with positive status applying immunohistochemistry (IHC) for the histological oral areas (gingival sites) including for short-term and long-term khat users

TP53 IHC	TP53 expression in gingival histological sections among short-term users (%)	TP53 expression in gingival histological sections among long-term users (%)
Strong	19/41 (46.34)	26/42 (61.90)
Moderate	22/41 (53.65)	16/42 (38.09)
Weak	0	0
None	0	0

## Discussion

In general, oral cancer is seen in 10.5 adults per 100,000. The incidence of oral cancer is higher among males than females. The risks of having oral cancer grow with age [21]. Cancer related to the lips and oral cavity begin from mutations in squamous cells within thin and flat cells of the oral space. As a result, the mutations in squamous cells increase the risk of developing squamous cell carcinomas (SCC). Cancer-affected cells might penetrate into deeper tissues with cancer expansion [22]. The morbidity rates of oral cancer become systematically higher because of people’s vulnerability to various risk factors.^6^ Moreover, SCC emerges in tissues to ensure the effect of absorbance partition of the toxic/carcinogenic elements between a body and its exterior. These effects are manifested in barrier-related somatic systems including the oral cavity, oesophagus, lungs, airways, cervix, vulva, urethra, and skin [8, 23, 24].

Khat has a variety of constituent compounds and hence chewing khat leads to different health outcomes. In most cases, the person’s gastrointestinal system along with the nervous system become subjected to the plant’s effects [12, 25]. In human anatomy, the nervous system secures autonomic (peripheral) somatic reactions such as constipation, urine retention and some acute cardiovascular issues. In the meantime, the central nervous system is responsible for psychological effects such as elevated alertness, attention, dependence, resistance, and tolerance [12]. Fresh khat leaves contain cathinone—a typical alkaloid that affects the central nervous system [26]. Some research has confirmed toxicity, mutagenicity, carcinogenicity, teratogenicity, and congenital effects from consuming khat, i.e., mammalian cells and animal models [27, 28].

Other studies have reported that long-term use of khat leaves might provoke increased emergence of oral cancer [11, 12]. Moreover, other health complications are also related to khat consumption including oesophageal cancer, insomnia, anorexia, gastric problems, depression, liver disease, and a variety of cardiac problems [28–32]. Studies also suggest that regular khat chewing intensifies blood pressure, increased heart rate, [33, 34] and elevates LDL cholesterol [35–37].

This research showed that OSCC develops in participants who used khat. During the study, seven somatic mutations in four of nine cancer-related genes were recorded among short-term users of khat. In addition, nine somatic mutations were identified in the group of long-term khat users in five of nine cancer-related genes. Participants from both groups were diagnosed with OSCC.

There were four common cancer-related genes associated with somatic mutations in two groups of respondents: *ARID1A*, *MLH1*, *PIK3CA*, and *TP53*. Epidemiological research has shown that mutation processes in the selected genes are attributed to higher risks of OSCC development [38–40].

Two landmark mutation processes were identified in *ARID1A* and *MLH1* related to DNA samples of long-term khat users, but a single landmark mutation was recorded in *MLH1* taken from short-term khat users. This was arranged by comparing the results to DNA samples from the control group. Another substitution mutation was noticed in *ARID2* and was related only to the group of long-term khat users. Noticeably, other studies assumed that systematic mutations in *ARID2* contribute to the oral tumorigenesis, which can be triggered by dysregulation of NF-κB signaling [41–43] and by active long-term tobacco smoking [44]. Nevertheless, TP53 protein’s expression was positive in all immunohistochemical oral areas, which reflected rates of OSCC development in both groups. Moreover, all affected participants had mutations in *TP53* genes—this is one of the most transforming genes in patients with OSCC diagnosis [45–48]. Histopathological tests also revealed poorly differentiated cells with unstable histological structure. This confirms the hypothesis that chewing khat, whether for short or long periods, eventually provoked OSCC.

### Implications for behavioural health

The mutagenicity and carcinogenicity effects from using and chewing khat plant have been insufficiently studied in Arabian and Asian cultures especially in short-term and long-term perspectives. The fresh findings derived in this study demonstrated a positive relation between khat use for short and long periods and somatic mutations: These were identified in nine cancer-related genes, thus increasing the risk of developing OSCC. The research shows that khat usage provoked high and systemic mutations in four to five of nine cancer-related genes. This was associated with provoking OSCC in short term khat users who were chewing the plant between 10 and 15 years; the same effect was seen among long-term khat users who were chewing for more than 15 years. In all cases, OSCC was the most prevalent health risk, which underscores the serious health threat of using khat for stimulation.

## Data Availability

The data that support the findings of this study are available on request from the corresponding author. The data are not publicly available due to privacy and ethical restrictions.
